# Dietary *Moringa oleifera* Leaves Enhance Biochemical Parameters Resulting in Increase in Egg Production of Layers

**DOI:** 10.1002/vms3.70916

**Published:** 2026-04-10

**Authors:** Voemesse Kokou, Nideou Dassidi, Lamboni Laré, Oyegunle Emmanuel Oke, Kokou Tona

**Affiliations:** ^1^ Centre of Excellence in Poultry Science University of Lome Lome Togo; ^2^ Institut Togolais de Recherche Agronomique Lomé Togo; ^3^ Université de Sarh Sarh Chad; ^4^ Department of Animal Physiology Federal University of Agriculture Abeokuta Nigeria

**Keywords:** biochemical parameters, egg‐type chick, egg production, *Moringa oleifera*

## Abstract

**Background:**

*Moringa oleifera* leaf meal (MOLM) supplementation is being investigated in the poultry industry for its nutritional and medicinal properties. However, its effects on birds’ performance, particularly egg production, are scarce.

**Objectives:**

This study was conducted to investigate the effect of different levels of MOLM on biochemical parameters and egg production of egg‐type chickens from day‐old to 55 weeks of age.

**Methods:**

A total of 450 day‐old chicks were assigned to three dietary treatments of basal diet supplemented with 0%, 1% and 3% MOLM, with 5 replicates of 30 birds each. Data on egg production were collected up to 55 weeks of age. Blood samples were collected from 15 birds per treatment via the jugular vein at 5, 15, 25, 35, 45, and 55 weeks of age for biochemical parameter analysis.

**Results:**

The results showed that feed intake was reduced in birds fed 1% and 3% of *M. oleifera*. Higher (*p* < 0.05) egg weights and lower triglyceride were recorded in the birds fed 3% of MOLM during the rearing period. However, plasma calcium, triglyceride level, and egg production were higher (*p* < 0.05) in birds fed 1% *M. oleifera* in the diet. Birds in the control group had lower (*p* < 0.05) average total cholesterol and higher (*p* < 0.05) feed conversion ratio than those of the other treatments.

**Conclusions:**

To conclude, the incorporation of 1% *M. oleifera* leaves in layer‐type diet for a long period resulted in an increase in blood calcium, triglyceride, and egg production, whereas 3% inclusion significantly increased egg weight but decreased triglyceride level.

## Graphical abstract

The incorporation of 1% *Moringa oleifera* leaves in layer‐type diet resulted in an increase in blood calcium, triglyceride, and egg production, whereas 3% inclusion increased egg weight but decreased triglyceride level. The enhanced egg production observed confirms *Moringa oleifera* as a natural feed additive for boosting commercial egg production

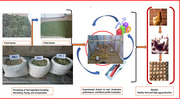

## 1 Introduction

Following the ban on antibiotics as growth promoters in commercial animal feed (Rabelo‐Ruiz et al. 2021; Castanon 2007), the poultry industry has focused on identifying suitable alternatives to maintain optimal health and production. Thus, herbs and a number of spices, which are known to contain biologically active substances (Bouyahya et al. 2020; Oni et al. 2024, 2025), have been considered nutritional supplements (Chodkowska et al. 2022; Iwiński et al. 2022). Although the mechanisms of action of these alternatives are not fully clear, recent research indicates that natural additives may inhibit pathogenic bacteria (Soyad and El‐Ghany 2020; Pham et al. 2022; Seidavi et al. 2022; Kumar et al. 2010; Dieumou et al. 2009). Their positive effect is also demonstrated on growth and egg productive performance, quality egg, and meat traits, as well as differential blood count and immunity response (Ghasemi et al. 2010; Navid et al. 2014; Nideou et al. 2017; Rabelo‐Ruiz et al. 2021).

To date, several studies have examined various parts of *Moringa oleifera*, but its leaves are most commonly used in poultry diets as leaf meal. *Moringa oleifera* leaves are a potent natural source of dietary antioxidants (Sreelatha and Padma 2009), antibacterial (Djakalia et al. 2011), minerals, and in particular essential amino acids (Ganatra et al. 2012; Aye and Adegun 2013; Kesharwani et al. 2014) and oestrogenic substances (Zade and Dabhadkar 2013). In the study of estrogenic activity in rats, Zade and Dabhadkar (2013) observed that *M. oleifera* induced vaginal opening and vaginal cornification and increased uterine weight in immature rats due to the presence of phytochemicals such as alkaloids, steroids, flavonoids, and phenolics.

Given their compounds and properties, *M. oleifera* leaves can be considered an ideal dietary supplement for the poultry industry to enhance growth, organ development, and overall production performance. Although preliminary studies are underway in different laboratories to study the effect of *M. oleifera* leaf meal on the performance of commercial layers, many of these studies have only focused on the growth (Melesse et al. 2011; Voemesse et al. 2018) and productive period of laying hens (Kakengi et al. 2007; Abou‐Elezz et al. 2011). There is a scarcity of studies available on the use of *M. oleifera* leaf meal as a phytogenic feed additive on the growth and productive periods of layer chicks. Thus, the aim of this study was to investigate the effect of *Moringa oleifera* on biochemical parameters and productive performance of egg‐type chicks from day‐old to 55 weeks of age

## 2 MATERIALS AND METHODS

### 2.1 Experimental Design

Four hundred and fifty Isa Brown day‐old chicks were used in the study and reared for 55 weeks of age. The day‐old chicks were assigned randomly to three treatment groups (T0, T1, T3) having five replicates of 30 birds each. The treatments were a basal diet containing 0% (T0), 1% (T1), and 3% (T3) of *M. oleifera* leaf meal (MOLM). Leaves harvested from famer's field were dried under air conditioning and hand‐crushed before using. All diets had similar levels of crude protein and metabolizable energy and met the requirements of the birds according to their stage of development (Table 1). The hens were supplied with feed and water ad libitum.

**TABLE 1:** Gross composition of experimental diet (%).

Feed composition according to age and group										
	Starter mash			Gower mash				Layer mash		
Feed staff	T0	T1	T3	T0	T1		T3	T0	T1	T3
Maize	56	55.62	54.87	54	53.6		52.85	58	57.5	56.75
Wheat bran	11	10.74	10.23	24	23.75		23.25	8	7.9	7.35
Fish meal	8	8	7.8	7	7		6.8	8	7.6	7.6
Soya seed	20	19.64	19.1	11	10.65		10.1	17	17	16.3
Concentrate	4	4	4	2	2		2	2	2	2
Oyster shell	1	1	1	2	2		2	7	7	7
*Moringa* leaves	0	1	3	0	1		3	0	1	3
Total	100	100	100	100	100		100	100	100	100
Calculated analysis										
E.M. (kcal/kg)	2962.50	2962.01	2962.24	2740.69	2740.03	2740.10		2835.23	2835.89	2835,29
Crude protein (%)	20.11	20.11	20,11	16.80	16.80	16.80		17.67	17.68	17.69
Calcium (%)	0.97	0.97	0.96	1.14	1.14	1.13		2.58	2.56	2.56
Phosphorus (%)	0.74	0.73	0.72	0.73	0.73	0.72		0.62	0.61	0.60
Lysine total (%)	1.09	1.08	1.06	0.87	0.87	0.84		0.96	0.94	0.92
Methionine total (%)	0.48	0.48	0.47	0.38	0.38	0.37		0.40	0.39	0.39
Meth. + cysteine (%)	0.72	0.71	0.70	0.60	0.60	0.58		0.61	0.60	0.58

Abbreviation: E.M., energy metabolizable.

### 2.2 Biochemical Analysis

At 5 weeks of age and every 10 weeks onwards, blood samples were collected from the wing vein of 15 birds per treatment (three per replication) in the morning into a blood storage tube without anticoagulant and were kept on ice in a cool container. The blood was allowed to coagulate and then centrifuged at 3000 rpm to obtain serum, which was stored at −20°C for analysis. Serum constituents were determined calorimetrically using kits according to Gindler and King (1972) for calcium, El‐Merzabani et al. (1977) for phosphorus, Fassati and Prencipe (1982) for triglyceride, and Allain et al. (1974) for cholesterol.

### 2.3 Production Parameter

Feed intake (FI) was recorded daily. Egg number (EN) and egg weight (EW) were recorded daily and used to calculate weekly egg production % (EP%) and egg mass (EM).

$$\mathrm{EP}\;\left(\%\right)=\frac{\mathrm{EN}\;\times 100\;}{\mathrm{Hen}-\mathrm{days}},$$

$$\mathrm{EM}\;=\frac{\mathrm{EN}\times \mathrm{EW}\;}{\mathrm{Period}\;\left(\mathrm{days}\right)}.$$

Feed conversion ratio (FCR; gram of feed per gram of egg) was calculated the following equation:

$$\mathrm{FCR}\;=\frac{\mathrm{FI}\;\left(\mathrm{gram}\;\mathrm{of}\;\mathrm{feed}/\mathrm{hen}/\mathrm{period}\right)\;}{\mathrm{EM}\;\left(\mathrm{gram}\;\mathrm{of}\;\mathrm{egg}/\mathrm{hen}/\mathrm{period}\right)}.$$

### 2.4 Statistical Analysis

Statistical analysis was performed using GraphPad Prism 5. One‐way analysis of variance (ANOVA) was used to evaluate the effects of *Moringa* leaves on blood chemistry values and productive performance. Tukey's HSD test was used to compare means. Prior to analysis, the data were checked for normality and homogeneity of variances to ensure compliance with the assumptions of ANOVA. The results were considered statistically significant at a confidence level of *p <* 0.05. All values were expressed as mean ± standard error of the mean (SEM).

The one‐way statistical model is given as follows:

*Yij* = *μ* + *Ti* + *Eij*, where *Yij* is the single observation, *μ* is the overall mean, *Ti* is the treatment effect, and *eij* is the random error.

## 3 Results

### 3.1 Blood Biochemical Serum Parameters

#### 3.1.1 Effect of MOLM on Phosphorus and Calcium Concentration

**FIGURE 1:**
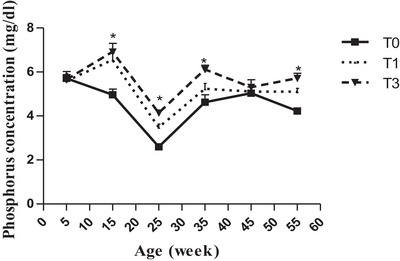
Phosphorus concentration (mg/dL) according to age and treatment. At each age, significant differences are indicated as follows: *p < 0.05. T0 (0% of MOLM), T1 (1% of MOLM), and T3 (3% of MOLM).

**FIGURE 2:**
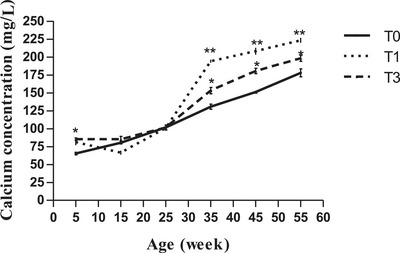
Calcium (mg/L) according to age and treatment. At each age, significant differences are indicated as follows: *p < 0.05; **p < 0.01. T0 (0% of MOLM), T1 (1% of MOLM), and T3 (3% of MOLM).

Serum phosphorus concentration generally increased across treatments (Figure 1). According to bird age, phosphorus showed fluctuations across all treatments, with higher values (*p* < 0.05) observed at weeks 15, 25, and 55 in T1 and T3 compared with T0 (Figure 1). However, serum calcium content varied significantly between treatments on the 15th and 25th week of age, but on week 35, 45 and continuously until the period investigated, birds in T1 had statistically higher values (*p* < 0.01) followed by those in T3 (*p* < 0.05) compared to control (Figure 2).

#### 3.1.2 Effect of MOLM on Triglyceride and Total Cholesterol Concentration

**FIGURE 3:**
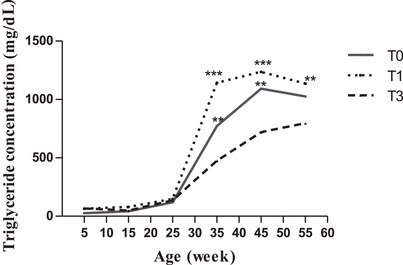
Triglyceride concentration (mg/dL) according to age and treatment. At each age, significant differences are indicated as follows: **p < 0.01; ***p < 0.001. T0 (0% of MOLM), T1 (1% of MOLM), and T3 (3% of MOLM).

**FIGURE 4:**
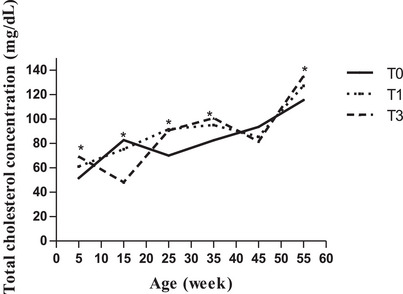
Total cholesterol concentration (mg/dL) according to age and treatment. At each age, significant differences are indicated as follows: *p < 0.05. T0 (0% of MOLM), T1 (1% of MOLM), and T3 (3% of MOLM).

In the lipid metabolites, triglyceride showed slightly increasing trends and was not affected by treatments on the 5th, 15th, and 25th week of age (Figure 3). From week 25 onward, a drastically higher level was detected, and between treatments, birds in T1 had higher values (*p* < 0.001; *p* < 0.01) for triglyceride level, while a marked decrease was found in the birds of M3 (*p* < 0.05) compared to those of the control group (Figure 3). Total cholesterol showed increasing trends across treatments (Figure 4). In general, total cholesterol mean values of the birds in T1 (89.34 ± 3.78 mg/dL) and T3 (87.60 ± 2.06 mg/dL) were higher (p˂0.05) than those of the control (82.69 ± 3.78 mg/dL).

#### 3.1.3 Effect of MOLM on Productive Performance

**FIGURE 5:**
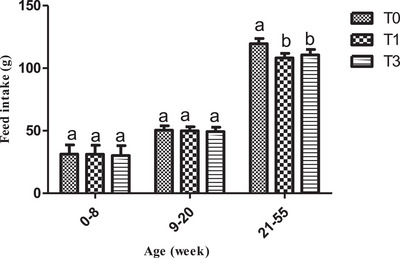
Feed intake according to treatment. At each treatment, means with different superscripts are significantly different (*p* < 0.05). T0 (0% of MOLM), T1 (1% of MOLM), and T3 (3% of MOLM).

**FIGURE 6:**
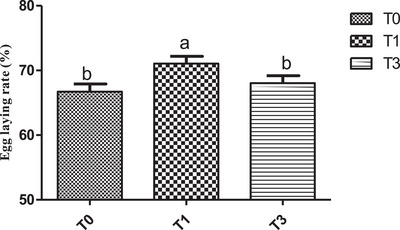
Egg laying rate (%) according to treatment. At each treatment, means with different superscripts are significantly different (*p* < 0.05). T0 (0% of MOLM), T1 (1% of MOLM), and T3 (3% of MOLM).

**FIGURE 7:**
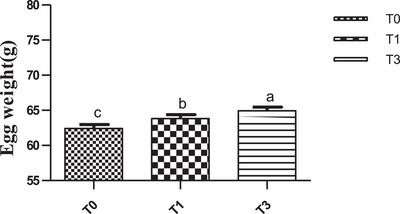
Egg weight (g) according to treatment. At each treatment, means with different superscripts are significantly different (*p* < 0.05). T0 (0% of MOLM), T1 (1% of MOLM), and T3 (3% of MOLM).

**FIGURE 8:**
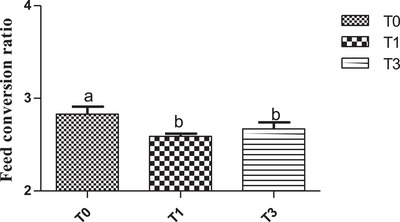
Feed conversion ratio according to treatment. At each treatment, means with different superscripts are significantly different (*p* < 0.05). T0 (0% of MOLM), T1 (1% of MOLM), and T3 (3% of MOLM).

Overall, feed intake values were similar between treatments during the rearing period but reduced (p˂0.05) in birds fed *M. oleifera* (Figure 5) during the egg production period. Figure 6 shows the mean egg‐laying rate across treatments. Egg production showed a clear upward trend in the T1 group and was statistically higher (*p* < 0.05) than in T0 and T3, whose values were similar (Figure 6). Across treatments, birds of T3 had a statistically higher average egg weight (*p* < 0.05) during the period investigated (Figure 7). With regard to the feed conversion ratio, values were comparable between T1 and T3 and were lower (p˂0.05) than those of the birds of T0 (Figure 8).

## 4 Discussion

The present study aimed to investigate the effect of *M. oleifera* on biochemical parameters and productive performance of layer chickens. Our study demonstrates clearly that the inclusion of *M. oleifera* leaves in layer‐type diet affected egg production as well as egg weight, and this effect was consistent with changes in some biochemical parameters.

It is noteworthy that all biochemical parameters analyzed increased during the egg production period, but not in the same way. In general, our results showed a significant increase in serum concentrations of calcium, phosphorus, triglycerides, and cholesterol in birds of T1 and T3, which also had a better feed conversion ratio. The improved results may be attributed to the essential nutrients of the leaves (Martínez et al. 2025) and also to their oestrogenic substances (Zade and Dabhadkar 2013). Earlier observations and studies had linked the increase in calcium during the egg production period to oestrogen secretion (Urist et al. 1958). This steroid hormone is involved in calcium metabolism regulation (Bar et al. 1996), especially during shell formation, lipid metabolism (Pallottini et al. 2008), and in the development of the reproductive organs. It is hypothesized that oestrogen affects Ca^2+^ transport in the duodenum by the up‐regulation of calcium channels (Van Cromphaut et al. 2003). Hwang et al. (2006) indicated that phytoestrogens seemed to act as antagonists in a high oestrogen environment and as agonists in a low oestrogen environment.

Thus, the reduction of egg production in the birds of T0 in the present study could be due to low endogenous oestrogen secretion, which adversely affected calcium and cholesterol levels. However, in birds fed with 1% of *M. oleifera*, endogenous estrogenic actions might have been amplified by phytoestrogens, leading to an increase in hepatic lipid metabolism and mobilization of calcium from bone for shell calcification. It is reported that hepatic lipid oxidation is decreased with oestrogen deficiency in rats (Paquette et al. 2009) and according to Bar et al. (1996), an injection of exogenous oestradiol increases plasma Ca^2+^ levels in laying hens.

Thus, hens in T1, which had higher triglycerides, would have used this metabolite to produce more energy by oxidation, which explains their egg production performance. In the same vein, Teteh et al. (2016) reported a higher value of triglyceride and egg production in layer hens fed 1% of *Moringa* leaves during 40 weeks of age. In contrast, 3% of *M. oleifera* in the layer diet exhibited a hypolipidemic effect in the present study, and this also corroborates the results of Teteh et al. (2016). It is thus hypothesized that the effects of long‐term incorporation of *Moringa* leaves in layer chicken diets on lipid metabolism are dose‐dependent. Indeed, birds of T3 might have been exposed to a high concentration of oestrogen due to the phytoestrogen, which acted as an antagonist and blocked oestrogen receptors, resulting in lowered triglyceride biosynthesis and mobilization of calcium and finally reduced egg production.

The high concentration of oestrogen (endogenous oestrogen and phytoestrogen) could also act by negative feedback on the hypothalamus, which may inhibit the release of Follicle Stimulating Hormon (FSH) and Luteinizing Hormon (LH) and subsequently reduce ovarian function. This is consistent with the findings of Teteh et al. (2016), who showed low weights of the ovarian and oviduct in laying hens fed 2% of *M. oleifera* during 40 weeks of age. Furthermore, the decrease of calcium and triglyceride levels in M3 may also be attributed to the presence of anti‐nutritional factors in *Moringa* leaves. Indeed, the leaves are reported to contain oxalate and saponins (Athira et al. 2021), which chelate calcium (Gupta et al. 1989) and inhibit intestinal dietary triglyceride uptake (Dong et al. 2007), respectively.

While leaf bioactive compounds stimulated egg production in T1, they affected egg size in T3. A possible explanation for the larger egg size in T3 is increased albumin. This interpretation is supported by our previous studies, which showed a significant increase in plasma albumin in birds fed with 3% of *M. oleifera* (Voemesse et al. 2019). This might have contributed to synthesizing more albumen and also explained their better feed conversion ratio compared to the birds of the control group.

## 5 Conclusion

It is concluded that *M. oleifera* leaves incorporated at 1% in the diet of egg‐type chickens increased plasma calcium, cholesterol, and triglyceride levels and egg production, while 3% reduced triglyceride and plasma calcium but increased egg weight. Therefore, *M. oleifera* can be used by farmers at 1% to enhance the production of birds’ eggs. However, further investigation is needed to elucidate the oestrogenic action of *M. oleifera* leaves on hen physiology by measuring reproductive hormone levels and the expression of related genes.

## Author Contributions

Voemesse Kokou: formal analysis, methodology, visualization, writing – original draft. Nideou Dassidi: conceptualization, data curation, investigation, writing – review and editing. Lamboni Laré: conceptualization, data curation, methodology, writing – review and editing. Oyegunle Emmanuel Oke: methodology, data curation, writing – review and editing. Tona Kokou: Project administration, supervision, visualization, writing – review and editing.

## Funding

The authors have nothing to report.

## Ethics Statement

The authors confirm that the ethical policies of the journal, as noted on the journal's author guidelines page, have been adhered to and the appropriate ethical review committee approval has been received (Approval No. 008/2021/BC‐BPA/FDS‐UL). The US National Research Council's guidelines for the Care and Use of Laboratory Animals were followed.

## Conflicts of Interest

The authors declare no conflicts of interest.

## Data Availability

The data that support the findings of this study are available on request from the corresponding author. The data are not publicly available due to privacy or ethical restrictions.
